# Dengue Virus Entry as Target for Antiviral Therapy

**DOI:** 10.1155/2012/628475

**Published:** 2012-03-04

**Authors:** Marijke M. F. Alen, Dominique Schols

**Affiliations:** Department of Microbiology and Immunology, Rega Institute for Medical Research, Katholieke Universiteit Leuven, 3000 Leuven, Belgium

## Abstract

Dengue virus (DENV) infections are expanding worldwide and, because of the lack of a vaccine, the search for antiviral products is imperative. Four serotypes of DENV are described and they all cause a similar disease outcome. It would be interesting to develop an antiviral product that can interact with all four serotypes, prevent host cell infection and subsequent immune activation. DENV entry is thus an interesting target for antiviral therapy. DENV enters the host cell through receptor-mediated endocytosis. Several cellular receptors have been proposed, and DC-SIGN, present on dendritic cells, is considered as the most important DENV receptor until now. Because DENV entry is a target for antiviral therapy, various classes of compounds have been investigated to inhibit this process. In this paper, an overview is given of all the putative DENV receptors, and the most promising DENV entry inhibitors are discussed.

## 1. Introduction

Dengue virus (DENV) is a single-stranded, positive-sense enveloped RNA virus of the *Flaviviridae* family that is transmitted by *Aedes aegypti* and *Aedes albopictus*. There exist 4 different serotypes of DENV. Each serotype shares around 65% of the genome, and, despite of the differences, each serotype causes nearly identical syndromes in humans and circulates in the same ecological niche [1]. Dengue virus causes clinical syndromes in humans, ranging from an acute self-limited febrile illness (dengue fever, DF) to a severe and life-threatening vascular leakage and shock (dengue hemorrhagic fever/dengue shock syndrome, DHF/DSS) [2, 3]. In the last decade, due to a decline of vector control efforts, DENV has reemerged in tropical areas and is considered as the most common arthropod-borne tropical disease that endangers an estimated 2.5 billion people [4, 5]. Every year, 50 million infections occur, including 500,000 hospitalizations for DHF, mainly among children, with a case fatality rate exceeding 5% in some areas. At present, diagnosis is largely clinical, treatment is supportive through hydration, and disease control is limited by eradication of the mosquito. Many efforts have been made in the search for a suitable vaccine, but the lack of an animal model and the need for a high immunogenicity vaccine against all four serotypes and a low reactogenicity are posing huge challenges in the dengue vaccine development [6, 7]. As there is no vaccine available, the search for antiviral products is imperative. Antivirals previously designed against flaviviruses have principally focused on inhibition of viral RNA replication. Ribavirin [8], mycophenolic acid [9], and adenosine analogues [10] are believed to act as inhibitors of the RNA-dependent RNA polymerase. Due to low efficacy of these types of compounds [9, 11, 12], more tolerable, highly potent DENV inhibitors are urgently needed. In the past few years, progression has been made in unraveling the host cell pathways upon DENV infection. It is proposed that viral epitopes on the surface of DENV can trigger cellular immune responses and subsequently the development of a severe disease. Therefore, these epitopes are potential targets for the development of a new class of antiviral products, DENV entry inhibitors. Inhibition of DENV attachment and entry into the host cell can inhibit immune activation. The cellular immune response is believed to play an important role in antibody-dependent enhancement (ADE). This is a phenomenon where cross-reacting nonneutralizing antibodies generated to the first DENV infection will recognize a heterologous DENV during a secondary infection with another serotype. The DENV-Ab complex enhances DENV access to Fc-receptor bearing cells [13, 14]. This results in the proliferation of T cells and the production of proinflammatory cytokines that have an indirect effect on the vascular endothelial cells leading to plasma leakage and DHF [3, 4, 15].

This paper will focus on the entry process of DENV and on all identified cellular DENV receptors. A better understanding of the role of the structural envelope protein would aid the research and development of entry inhibitors against flaviviruses. Inhibition of virus attachment is a valuable antiviral strategy because it forms the first barrier to block infection. Specific molecules preventing the interaction between the host and DENV envelope are discussed.

## 2. DENV Entry

### 2.1. Entry Process

**Figure 1:**
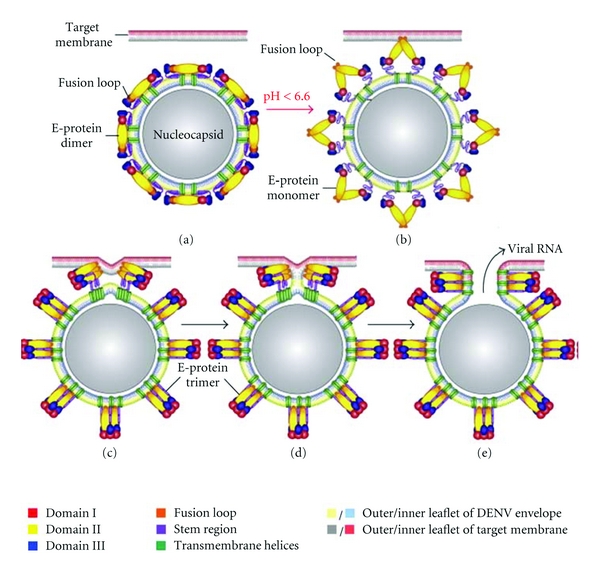
Schematic overview of the DENV membrane fusion process (modified figure from Stiasny et al., Amino Acids, 2009 [25]). (a) Prefusion conformation of the E-protein consists of homodimers on the virus surface. (b) Low endosomal pH triggers dissociation of the E-dimers into monomers which leads to the insertion of the fusion peptide with the endosomal target membrane. (c) A stable E-protein trimer is folded in a hairpin-like structure. (d) Hemifusion intermediate in which only the outer leaflets of viral and target cellular membranes have fused. (e) Formation of the postfusion E-trimer and opening of the fusion pore allow the release of the viral RNA into the cytoplasm.

The infectious entry of DENV in its target cells, mainly dendritic cells [16], monocytes, and macrophages, is mediated by the viral envelope glycoprotein E via receptor-mediated endocytosis [17]. The E-protein is the major component (53 kDa) of the virion surface and is arranged as 90 homodimers in mature virions [18]. Recent reports demonstrated that DENV enters its host cell via clathrin-mediated endocytosis [19, 20], comparable with other flaviviruses [21, 22]. Evidence for flavivirus entry via this pathway is based on the use of inhibitors of clathrin-mediated uptake, such as chlorpromazine. However, DENV entry via a nonclassical endocytic pathway independent from clathrin has also been described [23]. It seems that the entry pathway chosen by DENV is highly dependent on the cell type and viral strain. In case of the classical endocytic pathway, there is an uptake of the receptor-bound virus by clathrin-coated vesicles. These vesicles fuse with early endosomes to deliver their cargo into the cytoplasm. The E-protein responds to the reduced pH of the endosome with a large conformational rearrangement [24, 25]. The low pH triggers dissociation of the E-homodimer, which then leads to the insertion of the fusion peptide into the target cell membrane forming a bridge between the virus and the host. Next, a stable trimer of the E-protein is folded into a hairpin-like structure and forces the target membrane to bend towards the viral membrane, and eventually fusion takes place [24, 26, 27]. The fusion results in the release of viral RNA into the cytoplasm for initiation of replication and translation (Figure 1).

#### 2.1.1. Human Cell DENV Attachment and Receptors

Prior to fusion, DENV needs to attach to specific cellular receptors. Because DENV can infect a variety of different cell types isolated from different hosts (human, insect, monkey, and even hamster), the virus must interact with a wide variety of cellular receptors. In the last decade, several candidate attachment factor/receptors are identified (Table 1).

**(1) Immune Cells (Monocytes, Dendritic Cells, and Macrophages)**

Since 1977, monocytes are considered to be permissive for DENV infection [70]. More recent, phenotyping of peripheral blood mononuclear cells (PBMCs) from pediatric DF and DHF cases resulted in the identification of monocytes as DENV target cells [71]. First, it was believed that monocytes are important during secondary DENV infections during the ADE process, because of their Fc-receptor expression. The complex formed between the nonneutralizing antibody and the virus can bind to Fc-receptors and enhance infection in neighbouring susceptible cells [13, 14, 17]. There is evidence for the expression of a trypsin-sensitive receptor on monocytes facilitating DENV infection [72]. Later, it was shown that DENV can enter monocytes in a CD14-dependent manner, because lipopolysaccharide (LPS) can inhibit the infection [32]. After LPS binding, heat shock protein (HSP) 70 and HSP90 are clustered around CD14, preventing them from interacting with DENV [33]. This indicates that HSP70 and HSP90 are part of a receptor complex in monocytes.

**Figure 2:**
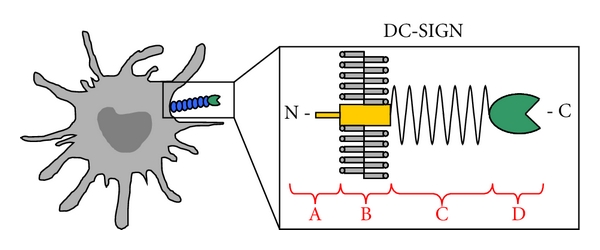
Structure of DC-SIGN. DC-SIGN, mainly expressed by human dendritic cells in the skin, is composed out of four domains: (A) cytoplasmic domain containing internalization signals, (B) transmembrane domain, (C) 7 or 8 extracellular neck repeats implicated in the oligomerization of DC-SIGN, and (D) carbohydrate recognition domain which can interact calcium-dependently with a variety of pathogens.

More detailed observation of the natural DENV infection changes the idea of monocytes being the first target cells. Following intradermal injection of DENV-2 in mice, representing the bite of an infected mosquito, DENV occurs to replicate in the skin [73]. The primary DENV target cells in the skin are believed to be immature dendritic cells (DCs) or Langerhans cells [16, 74–76]. Immature DCs are very efficient in capturing pathogens whereas mature DCs are relatively resistant to infection. The search for cellular receptors responsible for DENV capture leads to the identification of cell-surface C-type lectin DC-specific intercellular adhesion molecule 3-grabbing nonintegrin (DC-SIGN; CD209) [34, 35, 61, 77]. DC-SIGN, mainly expressed by immature DC as a tetramer, is a member of the calcium-dependent C-type lectin family and is composed out of four domains: a cytoplasmic domain responsible for signaling and internalization due to the presence of a dileucine motif, a transmembrane domain, seven to eight extracellular neck repeats implicated in the oligomerization of DC-SIGN, and a carbohydrate recognition domain (CRD) (Figure 2) [78]. The CRD recognizes high-mannose N-glycans and fucose-containing blood group antigens [79, 80]. Importantly, DC-SIGN can bind a variety of pathogens like human immunodeficiency virus (HIV) [81], hepatitis C virus (HCV) [82], Ebola virus [83], and several bacteria, parasites, and yeasts [84]. Many of these pathogens have developed strategies to manipulate DC-SIGN signaling to escape from an immune response [84]. Following antigen capture in the periphery, DCs maturate by upregulation of the costimulatory molecules and migrate to secondary lymphoid organs. Activated DCs are stimulators of naive T cells and they initiate production of cytokines and chemokines [85]. Inhibition of the initial interaction between DENV and DC could prevent an immune response. DC-SIGN could be a target for antiviral therapy by interrupting the viral entry process.

Besides DC, macrophages play a key role in the immunopathogenesis of DENV infection as a source of immunomodulatory cytokines [86]. Recently, Miller et al. showed that the mannose receptor (MR; CD206) mediates DENV infection in macrophages by recognition of the glycoproteins on the viral envelope [36]. MR is also present on monocyte-derived DC (MDDC), and anti-MR antibodies can inhibit DENV infection, although to a lesser extent than anti-DC-SIGN antibodies do [62]. MR differs from DC-SIGN in ligand specificity and acts as an internalization receptor for DENV instead of an attachment factor. Another C-type lectin, CLEC5A (C-type lectin domain family 5, member A) expressed by human macrophages can also interact with DENV and acts as a signaling receptor for the release of proinflammatory cytokines [37]. However, whereas the DC-SIGN-DENV interaction is calcium-dependent, CLEC5A binding to its ligand is not dependent on calcium. Mannan and fucose can inhibit the interaction between CLEC5A and DENV, indicating that the interaction is carbohydrate-dependent [37]. However, a glycan array demonstrated that there is no binding signal between CLEC5A and N-glycans of mammals or insects [87]. The molecular interaction between CLEC5A and DENV remains to be elucidated.

Immune cells, in particular dendritic cells, are the most relevant cells to use in the discovery of antiviral drugs against dengue virus, but the isolation of these cells and the characterization is labour intensive and time consuming.

**(2) Liver Cells**

The liver is an important target organ of dengue, in particular in DHF and DSS, because liver enzymes are usually elevated [88] and apoptosis of hepatocytes has been reported [89]. The interaction of DENV with liver cells has been studied.

Heparan sulfate (HS), the most ubiquitous member of the glycosaminoglycan (GAG) family, present on human hepatocytes, is described as a putative receptor for DENV [28, 29, 38, 43]. HS is also expressed by Vero cells, CHO cells, and BHK cells which are widely used in the study of dengue virus infection because of the easy cell growth conditions. HS very often acts as an attachment factor to concentrate the virus on the cell surface to facilitate binding to a second receptor. However, the contribution of HS to internalize DENV appears to vary in a serotype-specific manner [39, 90]. In Vero cells, a putative glycoprotein coreceptor is characterized of 74 kDa binding DENV-4 in a carbohydrate-dependent manner [30]. Another carbohydrate molecule characterized to interact with all four serotypes of DENV in BHK cells and insect cells is the terminal disaccharide of a glycosphingolipid, neolactotetraosylceramide [31, 44].

Besides HS [38, 39], glucose-regulated protein 78 (GRP78) is identified as a possible liver receptor in hepatocytes [40]. Wati et al. showed that GRP78 is also upregulated in DENV-infected monocytes and acts as a chaperone for viral-protein production during DENV infection [91]. Liver cells are important target cells during dengue virus infection, and the liver cell line Huh-7 has easy growth conditions. In general, liver cells are not widely used for studying dengue virus infection, but liver cells have more clinical relevance in contrast to monkey cells (Vero) or hamster cells (BHK) and should get more attention to use in screening discovery programs for antiviral drugs.

**(3) Endothelial Cells**

Liver/lymph node-specific ICAM-3 grabbing nonintegrin (L-SIGN) is a DC-SIGN-related transmembrane C-type lectin expressed on endothelial cells in liver, lymph nodes, and placenta [92, 93]. Similar to DC-SIGN, L-SIGN is a calcium-dependent carbohydrate-binding protein and can interact with HIV [92], HCV [82], Ebola virus [83], West Nile virus [94], and DENV [35]. Although endothelial cells [95] and liver endothelial cells [89] are permissive for DENV and L-SIGN-expression makes unsusceptible cells susceptible for DENV infection, the *in vivo* role for L-SIGN in DENV entry remains to be established. Upregulation of *β*3 integrin has been observed following DENV infection in human endothelial cells [42], and DENV entry is highly dependent on the expression of *β*3 integrin. This indicates that *β*3 integrin can act as an important secondary receptor for DENV entry in endothelial cells.

#### 2.1.2. Mosquito Cell DENV Attachment and Receptors

DENV entry into mosquito cells is poorly understood. Previously, electron microscopic studies in the *Aedes Albopictus* mosquito cell line, C6/36, have shown that DENV penetrates directly into the cytoplasm by fusion at the plasma membrane [96]. In contrast, experiments concentrating on cell fusion of mosquito cells and virus inhibition with acidotropic agents have provided evidence of viral uptake through receptor-mediated endocytosis [97]. Recently, according to overlay protein-binding assays, two surface proteins on C6/36 cells with molecular masses 80 en 67 kDa have been demonstrated to interact with all four serotypes of DENV [98]. This is in contrast with other reports, where a surface protein of 45 kDa was identified as a receptor for DENV-4 in C6/36 cells [46] which was later designated as a heat-shock-related protein (HSP related) [47]. Also, the 37/67 kDa protein was identified as the laminin receptor expressed by C6/36 cells and hepatocytes [41, 45]. However, the binding capacity of DENV to interact with the laminin receptor is serotype-specific (only DENV-3 and DENV-4) and cell-type-dependent (only detected in larvae cells and not in adult mosquito cells). Recently, prohibitin is characterized as a DENV-2 receptor in insect cells [48]. However, it is unclear if this conserved eukaryotic protein plays a role in DENV infection in mammalian cells.

## 3. The DENV Envelope

The DENV E-protein induces protective immunity, and flavivirus serological classification is based on its antigenic variation. During replication, the virion assumes three conformational states: the immature, mature, and fusion-activated form. In the immature state, the E-protein is arranged as a heterodimer and generates a “spiky” surface because the premembrane protein (prM) covers the fusion peptide. In the Golgi apparatus, the virion maturates after a rearrangement of the E-protein. The E-heterodimer transforms to an E-homodimer and results in a “smooth” virion surface. After a furin cleavage of the prM to pr and M, the virion is fully maturated and can be released from the host cell. Upon fusion, the low endosomal pH triggers the rearrangement of the E-homodimer into a trimer [99].

**Figure 3:**
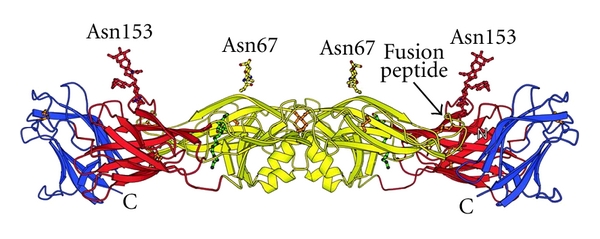
Location of the 2 N-glycans on the envelope protein of DENV. The DENV E-protein dimer carries 2 N-glycans on each monomer at Asn67 and Asn153. *β*-strands are shown as ribbons with arrows, *α*-helices are shown as coiled ribbons. Thin tubes represent connecting loops. Domain I is shown is red, domain II is shown in yellow and contains the fusion peptide near Asn153. Domain III is shown in blue. Disulfide bridges are shown in orange. In green, the ligand N-octyl-D-glucoside is shown, which interacts with the hydrophobic pocket between domains I and II (modified figure from Modis et al., PNAS, 2003) [100].

The E-protein monomer is composed out of *β*-barrels organized in three structural domains (Figure 3). The central domain I contains the aminoterminus and contains two disulphide bridges. Domain II is an extended finger-like domain that bears the fusion peptide and stabilizes the dimer. This sequence contains three disulphide bridges and is rich in glycine. Between domain I and domain II is a binding pocket that can interact with a hydrophobic ligand, the detergent *β*-N-octyl-glucoside. This pocket is an important target for antiviral therapy because mutations in this region can alter virulence and the pH necessary for the induction of conformational changes. The immunoglobulin-like domain III contains the receptor-binding motif, the C-terminal domain, and one disulphide bond [100, 101]. Monoclonal antibodies recognizing domain III are the most efficient of blocking DENV [102, 103] and this domain is therefore an interesting target for antiviral therapy.

Because DC-SIGN is identified as a receptor for DENV in primary DC in the skin and DC-SIGN recognizes high-mannose sugars, carbohydrates present on the E-protein of DENV could be important for viral attachment. The E-protein has two potential glycosylation sites: Asn67 and Asn153. Glycosylation at Asn153 is conserved in flaviviruses, with the exception of Kunjin virus [104] and is located near the fusion peptide in domain II [100, 101] (Figure 3). Glycosylation at Asn67 is unique for DENV [101]. The glycosylation at Asn67 is demonstrated to be essential for infection of MDDC, indicating an interaction between DC-SIGN and the glycan at Asn67 [105, 106]. Generally, the function of glycosylation of surface proteins is proper folding of the protein, trafficking in the endoplasmic reticulum, interaction with receptors, and influencing virus immunogenicity [107]. 

There are some contradictions in terms of necessity of glycosylation of Asn67 and Asn153 during DENV viral progeny. Johnson et al. postulated that DENV-1 and DENV-3 have both sites glycosylated and that DENV-2 and DENV-4 have only one N-glycan at Asn-67 [108]. In contrast, a study comparing the number of glycans in multiple isolates of DENV belonging to all four serotypes led to the consensus that all DENV strains have two N-glycans on the E-protein [109]. Nevertheless, mutant DENV lacking the glycosylation at Asn153 can replicate in mammalian and insect cells, indicating that this glycosylation is not essential for viral replication [105, 110]. However, there is a change in phenotype because ablation of glycosylation at Asn153 in DENV is associated with the induction of smaller plaques in comparison to the wild type virus [105]. Asn153 is proximal to the fusion peptide, and therefore deglycosylation at Asn153 showed also an altered pH-dependent fusion activity and displays a lower stability [111, 112].

DENV lacking the glycosylation at Asn67 results in a replication-defective phenotype, because this virus infects mammalian cells weakly and there is a reduced secretion of DENV E-protein. Replication in mosquito cells was not affected, because the mosquito cells restore the N-glycosylation at Asn67 with a compensatory site-mutation (K64N) generating a new glycosylation site [105, 113]. These data are in contrast with other published results, where was demonstrated that DENV lacking the Asn67-linked glycosylation can grow efficiently in mammalian cells, depending on the viral strain and the amino acid substitution abolishing the glycosylation process [110]. A compensatory mutation was detected (N124S) to repair the growth defect without creating a new glycosylation site. Thus, the glycan at Asn67 is not necessary for virus growth, but a critical role for this glycan in virion release from mosquito cells was demonstrated [110].

Virions produced in the mosquito vector and human host may have structurally different N-linked glycans, because the glycosylation patterns are fundamentally different [109, 114]. N-glycosylation in mammalian cells is often of the complex type because a lot of different processing enzymes could add a diversity of monosaccharides. Glycans produced in insect cells are far less complex, because of less diversity in processing enzymes, and usually contain more high-mannose and pauci-mannose-type glycans. DC-SIGN can distinguish between mosquito and mammalian cell-derived alphavirus [115] and West Nile virus [94], resulting in a more efficient infection by a mosquito-derived virus, but this was not the case for DENV [109].

## 4. DENV Entry Inhibitors

### 4.1. Fusion Inhibitors

By docking experiments and physicochemical algorithms using the structural data of the E-protein, small molecules and peptides targeting the hydrophobic pocket are characterized as entry inhibitors of DENV (Table 2) [49–51]. Nicholson et al. showed that two peptide entry inhibitors, DN59 and 1OAN1, could inhibit ADE *in vitro*, indicating that entry inhibitors could prevent development of the more severe disease outcome of dengue, DHF/DSS [116]. Tetracycline derivates have been shown to interact with the hydrophobic pocket of the E-protein (Figure 3) and, due to steric hindrance, prevent conformational rearrangements of the E-protein and subsequently prevent viral fusion [52]. A derivate of the antibiotic doxorubicin, SA-17, has a structure partially similar to tetracycline. SA-17 has been demonstrated to have antiviral activity against DENV serotype 1, 2, and 3 in Vero and C6/36 cells and interferes with viral entry by binding to the hydrophobic pocket of the E-protein without being virucidal [53]. Recently, two fusion assays using C6/36 cells have been optimized to examine the antifusion activities of a variety of compounds. NITD448, selected in docking experiments, was demonstrated to inhibit DENV-2 fusion by binding to the hydrophobic pocket of the E-protein [54]. All these compounds can serve as lead compounds for further drug discovery and for further elucidation of the entry process of DENV.

### 4.2. Glycosidase Inhibitors

Because of the risk of ADE, it is very important to achieve maximal protection to the same extent against all four serotypes with one drug or vaccine. Inhibitors targeting host cell processes, as glycosylation processes, are interesting targets and could overcome this problem. We will further focus on some *α*-glycosidase inhibitors that affect the modification of N-glycosylation of the viral proteins in the endoplasmic reticulum (ER).

The two lead compounds in inhibiting glycoprotein folding are imino sugars deoxynojirimycin (DNJ) and castanospermine (CSP) which mimic glucose (reviewed in [117]). CSP is a natural alkaloid derived from the black bean and is water soluble. CSP inhibits all four DENV serotypes by reducing the number of secreted particles, due to inappropriate glycoprotein folding, and by decreasing the infectivity of the secreted DENV particles [55, 56]. DNJ exerts the same mechanism of action as castanospermine [56]. Because of the low efficacy and cytotoxic effects, the development of imino sugars is limited. Alkylated iminocyclitol derivates, containing an imino sugar head group and an N-alkyl side chain, proved to be more potent against DENV-2 and less cytotoxic than DNJ [58]. N-alkylated derivates of DNJ (N-nonyl-DNJ (NN-DNJ)) have been shown to have increased antiviral potency compared to DNJ, but cytotoxic effects were also increased [57, 118]. However, NN-DNJ and a CSP derivate both reduced significantly viremia in a dengue fever mouse model [119]. Further optimization of the chemical structure of the imino sugar DNJ leads to the production of N-pentyl-(1-hydroxycyclohexyl)-DNJ (OSL-9511), an iminocyclitol with a DNJ head group, which showed reduced cytotoxicity and retained antiviral activity against DENV [59]. To improve the antiviral efficacy, a hydroxyl group was removed and an oxygen atom was added. This resulted in a new compound, CM-9-78, with exerted high anti-DENV activity and very low cytotoxicity [60]. Recently, the compound CM-9-78 and another variant CM-10-78 were tested *in vivo* and were shown to reduce viremia modestly by 2-fold. To improve the antiviral efficacy *in vivo*, a combination therapy was tested with ribavirin, a compound with a different antiviral mechanism of action. Whereas ribavirin by itself did not reduce viremia [119], combination of CM-10-78 and ribavirin demonstrated a clear enhancement in the reduction of viremia [120].

To conclude, there is a limited use of glycosidase inhibitors because of their toxicity and low specificity, but these compounds indeed help to understand the process of the E-protein glycosylation. In the last decade, not much progression has been made in the development of inhibitors targeting host glycosidase enzymes by biochemical modifications, but combination with other classes of inhibitors seems to achieve the best antiviral efficacy.

### 4.3. Carbohydrate-Binding Agents (CBAs)

**Figure 4:**
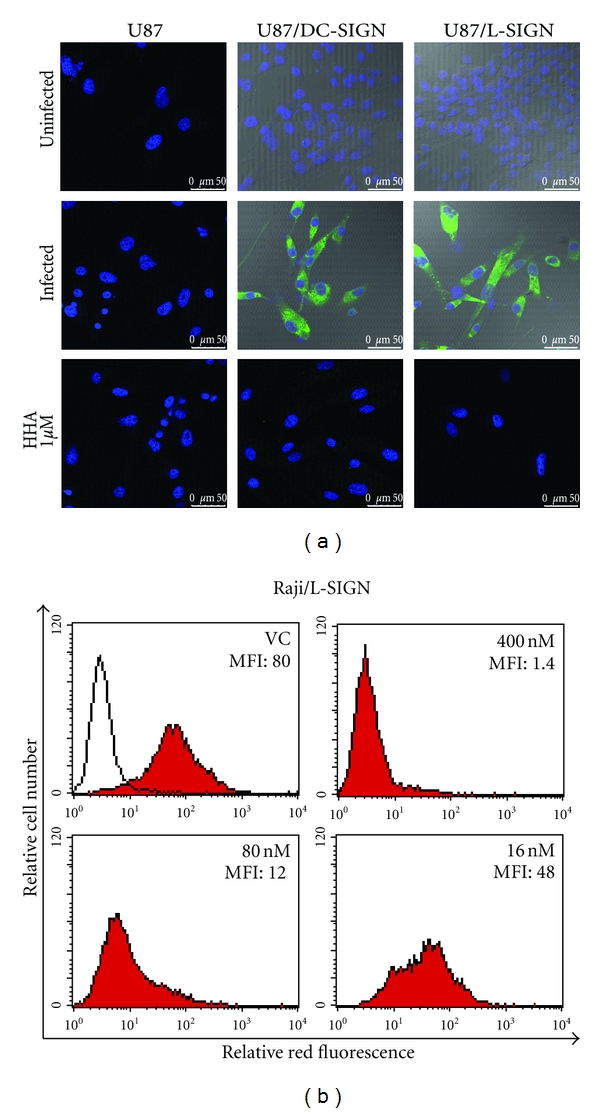
Antiviral activity of HHA in DC-SIGN and L-SIGN transfected cell lines. (a) U87, U87/DC-SIGN, and U87/L-SIGN cells were infected with DENV-2 in the presence or absence of HHA. DENV-2 infection was analyzed by confocal microscopy using specific DENV antibodies as previously described [62]. The cell nucleus is shown in blue (Dapi) and DENV-2 infected cells are shown in green (anti-DENV Ab + Alexa 488). (b) Raji/L-SIGN cells were infected with DENV-2 in the presence or absence of HHA, and viral infection was analyzed by flow cytometry using specific DENV antibodies. The open histogram represents uninfected cells. The upper left red histogram shows the virus control (VC), and the mean fluorescence of intensity (MFI) is indicated in each panel. HHA was added at different concentrations (400–80–16 nM), and the MFI shows the dose-dependent inhibition of the DENV-2 infection in Raji/L-SIGN cells. Comparable antiviral activity of HHA in Raji/DC-SIGN cells was obtained [61].

The CBAs form a large group of natural proteins, and they can be isolated from different organisms. Concanavalin A, isolated from the Jack bean, binds to mannose residues and wheat germ agglutinin (WGA) binds to N-acetylglucosamine (Glc-NAc) residues. Both compounds can reduce DENV induced plaque formation in BHK cells [43]. A competition assay, using mannose, proved that the inhibitory effect of Con A was due to binding *α*-mannose residues on the viral protein, because mannose successfully competed with Con A [43]. Recently, three plant lectins, *Hippeastrum hybrid* (HHA), *Galanthus nivalis* (GNA), and *Urtica dioica* (UDA), isolated from the amaryllis, snowdrop, and stinging nettle, respectively, have been shown to inhibit DENV-2 infection in Raji/DC-SIGN cells [61]. Binding studies revealed that the CBAs act during the adsorption phase of the virus to the host cell. HHA and GNA have been shown to interact with mannose-residues [121, 122], and UDA can recognize specifically Glc-NAc residues [123]. Mannose and Glc-NAc molecules are present in the backbone of the high-mannose type glycans on the viral envelope protein. Because DC-SIGN can also recognize these sugar molecules, the interaction between DC-SIGN and DENV E-glycoprotein is disrupted by HHA, GNA, and UDA. DC-SIGN, present on DC in the skin [16], is important during the first steps of a natural infection and thus forms an important target to focus on. The antiviral activity profile of the CBAs has been extended using different cell types. Recently, the antiviral activity of HHA, GNA, and UDA has been demonstrated in primary MDDC against all four DENV serotypes, and, importantly, the potency of the three CBAs was much higher in MDDC than in DC-SIGN transfected cell lines, such as Raji/DC-SIGN [62]. Raji cells and U87 cells transfected with L-SIGN, a DC-SIGN-related receptor, can be infected with DENV and this infection can also be inhibited with the three plant lectins (Figure 4 and unpublished data). However, since plant lectins are expensive to isolate in large quantities and not orally bioavailable, the search for nonpeptidic small molecules is necessary. PRM-S is a highly soluble nonpeptidic small-size carbohydrate-binding antibiotic and proved to inhibit DENV-2 in MDDC [62]. These data indicate that targeting the initial interaction between the N-glycans on the DENV envelope and the host cell is promising and that the CBAs have broad spectrum antiviral activity.

### 4.4. Heparan Mimetics

Because HS is a putative receptor for DENV, it is interesting to target the E-protein-HS interaction with soluble GAGs and other highly charged polyanions mimicking HS to prevent DENV entry (Table 2). GAG and heparin, a more highly sulfated protein than HS, can prevent binding of DENV to Vero cells and BHK cells [28]. Domain III of the E-protein is responsible for the interaction with HS [124]. It has been widely assumed that domain III is conserved within each DENV serotype and it is a good target for vaccines, because it contains epitopes recognized by neutralizing antibodies [102, 103].

The pharmaceutical product suramin, a small polyanion mimicking the structure of HS, and persulfated GAGs can bind to the polyanion-binding site of the DENV E-protein [125] and can inhibit DENV infection. Pentosan polysulfate (PPS) and the sulfated polysaccharide PI-88, which are currently in clinical trials for antitumor activity, inhibit DENV-2 infection in BHK cells. In IFN-*α*/*γ* receptor knock-out mice, a mouse model for DENV, PI-88 demonstrated an increase in survival time [63] whereas suramin and PPS did not show a beneficial effect *in vivo*.

Fucoidan, a sulfated polysaccharide isolated from marine alga, has specifically antiviral activity against DENV-2 in BHK cells and not against the other serotypes [64]. This is in agreement with others, where was demonstrated that sulfated polysaccharides from red seaweeds, carrageenan, and DL-galactan had antiviral activity against DENV-2 and DENV-3 but a very weak and no antiviral activity against DENV-4 and DENV-1, respectively, in human hepatocytes and Vero cells. The polysaccharides were not inhibitory in mosquito cells. Together with the fact that sulfated galactomannans are proved to be inhibitors of DENV-1 in C6/36 cells [65], these data indicate that the antiviral activity of sulfated polysaccharides is serotype- and cell-type-dependent [66].

Heparin analogues often have anticoagulant activities and this forms a major restriction factor for their use as antiviral product. Thus the search for polysaccharides with fewer side effects is imperative. DL-galactan from red seaweed lacks cytotoxic effects and anticoagulant properties and exhibits a high antiviral activity against DENV-2 [126]. Next, two *α*-D-glucans were isolated from a widely used Chinese herb with several therapeutic activities. These two polysaccharides exhibit anti-DENV-2 activity in BHK cells and sulfated derivates of one of the compounds proved even to be more potent [68]. This is in accordance with previous findings demonstrating that the antiviral activity of polysaccharides increases with molecular weight and degree of sulfation [28, 125]. 

There are some contrasting data concerning the antiviral activity of dextran sulfate. Dextran sulfate with molecular weight 8000 Da (DS8000) has been shown to have antiviral activity against DENV-2 in human hepatocytes and Vero cells [66]. This is in contrast with our data, where DS5000 had no antiviral activity against DENV-2 in Raji/DC-SIGN cells and Vero cells [61]. High molecular weight DS (MW = 500,000 Da) had no antiviral activity against DENV in Vero cells [28, 61], but recently this compound had been shown to inhibit DENV-2 in human Raji/DC-SIGN cells [61]. These data reinforce the idea that the entry process and thereby the antiviral activity of sulfated polyanions is cell-type- and serotype-dependent.

Another sulfated compound is the antiadhesive compound p-sulfoxy-cinnamic acid, zosteric acid, derived from a marine eelgrass. It showed to be nontoxic and inhibitory against all four serotypes in LLC-MK2 cells [69]. It has been shown that this compound promotes inappropriate virus-cell attachment and prevents virus entry.

In general, binding studies revealed that polysaccharides act during virus adsorption and internalization [66, 127]. The mechanism of action of carrageenan is by inhibition of a postadsorption process, namely, the release of the viral nucleocapsid into the cytoplasm, probably due to the interaction with the E-protein [67]. The antiviral effect of HS mimetics is probably due to steric hindrance and the negative charged sulfate groups, but there is a dose-limiting effect due to their anticoagulant activity. The antiviral activity of sulfated polyanions is cell-type- and serotype-dependent and thus not suitable for further clinical testing. 

## 5. Conclusion

DENV is able to infect many types of host cells and this resulted in the identification of several putative DENV receptors. DCs in the skin are believed to be the first target cells, and therefore DC-SIGN is assumed to be the most important DENV receptor until now. The unraveling of the entry process of DENV into the host cell and the recent progresses in virtual screening and docking techniques have lead to the development of a new class of DENV inhibitors, entry inhibitors. This class of compounds has great potential to be used either alone or in combination therapy with viral replication inhibitors. It has been shown that entry inhibitors can prevent ADE in human cells and subsequently immune activation [116]. This indicates a very important feature for further development of entry inhibitors and for future clinical studies.

## Figures and Tables

**Table 1:** Susceptible cell types for DENV infection and putative DENV receptors.

Species	Cell type	Cell description	DENV receptor(s)	References
*Monkey*	Vero	Kidney epithelium cells	Heparan sulfate (HS)	[28, 29]
			Glycoprotein (74 kDa)	[30]
	LLC-MK2	Kidney cells	Glycosphingolipid	[31]
*Human*	Monocytes	Primary myeloid cells	CD14/LPS	[32]
			HSP70/HSP90	[33]
			Fc-receptor	[13, 14, 17]
	Dendritic cells	Primary myeloid cells	DC-SIGN	[34, 35]
	Macrophages	Primary myeloid cells	Mannose receptor	[36]
			CLEC5A	[37]
	Huh	Hepatocytes	HS	[38, 39]
	HepG2	Hepatocytes	Laminin receptor, GRP78, HS	[40, 41]
	HMEC-1	Dermal endothelium	*β*3 integrin	[42]
*Hamster*	BHK	Kidney fibroblast cells	HS	[43]
			Glycosphingolipid	[44]
	CHO	Ovary cells	HS	[28, 29]
*Insect*	C6/36	*A. albopictus* larvae cells	Laminin receptor (37/67 kDa)	[45]
			HSP related (45 kDa)	[46, 47]
			Prohibitin (35 kDa)	[48]
	CCL-125	*A. Aegypti *larvae cells	Prohibitin (35 kDa)	[48]
	AP-61	*A. pseudoscutellaris* larvae cells	Glycosphingolipid	[31]

**Table 2:** Overview of all described DENV entry inhibitors.

Class	Compound	Serotype*	Cell line	References
*Fusion inhibitors*	1OAN1	DENV-2	LLC-MK2	[49]
	DN59	DENV-2	LLC-MK2	[50]
	Compound 6	DENV-1-4	A549, BHK	[51]
	Tetracycline derivates	DENV-2	BHK	[52]
	Doxorubicin derivate	DENV-1, -2, -3	Vero, C6/36	[53]
	NITD448	DENV-2	BHK, C6/36	[54]

*Glycosidase inhibitors*	Castanospermine	DENV-1-4	BHK	[55, 56]
		DENV-2	Huh-7	[55]
	DNJ	DENV-1	Mouse neuro 2a cells	[56]
	NN-DNJ	DENV-2	BHK	[57]
	Alkylated iminocyclitol	DENV-2	BHK	[58]
	OSL-9511	DENV-2	BHK	[59]
	CM-9-78	DENV-2	BHK	[60]

*CBAs*	Con A, WGA	DENV-2	BHK	[43]
	HHA, GNA, UDA	DENV-1-4	Raji/DC-SIGN, MDDC	[61, 62]
		DENV-1-4	Huh-7, U87/DC-SIGN	Unpublished results
		DENV-1-4	Raji/L-SIGN, U87/L-SIGN	Unpublished results
	PRM-S	DENV-2	MDDC	[62]

*Heparan mimetics*	GAG	DENV-2	Vero	[28]
	Heparin	DENV-2	Vero	[28, 29]
		DENV-2	BHK	[43]
		DENV-2	Hepatocytes	[38]
	Suramin	DENV-2	Vero, BHK	[28, 63]
	PI-88	DENV-2	BHK and in mice	[63]
	PPS	DENV-2	BHK	[63]
	Fucoidan	DENV-2	BHK	[64]
	Sulfated galactomannan	DENV-1	C6/36	[65]
	DL-galactan	DENV-2, -3	Vero, HepG2	[66]
	Carrageenan	DENV-2, -3	Vero, HepG2	[66, 67]
	*α*-D-glucan	DENV-2	BHK	[68]
	Dextran sulfate 8000	DENV-2	Hepatocytes, Vero	[66]
	DS (MW > 500,000 Da)	DENV-2	Raji/DC-SIGN	[61]
	Zosteric acid	DENV-1-4	LLC-MK2	[69]

*Serotype: The serotype mentioned is the serotype that has been tested and found susceptible to inhibition by the compound. Non-mentioned serotypes were not tested or could not be inhibited by the compound.
